# Essential Oil, Extracts, and Sesquiterpenes Obtained From the Heartwood of *Pilgerodendron uviferum* Act as Potential Inhibitors of the *Staphylococcus aureus* NorA Multidrug Efflux Pump

**DOI:** 10.3389/fmicb.2019.00337

**Published:** 2019-02-26

**Authors:** Javier Espinoza, Alejandro Urzúa, Loreto Sanhueza, Mariana Walter, Paola Fincheira, Patricia Muñoz, Leonora Mendoza, Marcela Wilkens

**Affiliations:** ^1^Laboratorio de Ecología Química, Departamento de Ciencias Químicas y Recursos Naturales, Universidad de La Frontera, Temuco, Chile; ^2^Centro de Excelencia en Investigación Biotecnológica Aplicada al Medio Ambiente, Universidad de La Frontera, Temuco, Chile; ^3^Laboratorio de Química Ecológica, Departamento de Ciencias del Ambiente, Universidad de Santiago de Chile, Santiago, Chile; ^4^Nucleo de Química y Bioquímica, Facultad de Estudios Interdisciplinarios, Universidad Mayor, Santiago, Chile; ^5^Laboratorio de Bioinorgánica SMATC, Departamento de Química de los Materiales, Universidad de Santiago de Chile, Santiago, Chile; ^6^Laboratorios de Biotecnología y Nanobiotecnología Ambiental, Departamento de Ciencias Químicas y Recursos Naturales, Universidad de La Frontera, Temuco, Chile; ^7^Laboratorio de Micología, Departamento de Química de los Materiales, Universidad de Santiago de Chile, Santiago, Chile; ^8^Laboratorio de Microbiología Básica y Aplicada, Departamento de Biología, Universidad de Santiago de Chile, Santiago, Chile

**Keywords:** antibiotic resistance, efflux pump, NorA, efflux pumps inhibitors, sesquiterpenes, 15-copaenol

## Abstract

*Staphylococcus aureus* is a serious human pathogen that is highly adaptive to environmental conditions and rapidly develops antibiotic resistance. The use of efflux pumps to reduce antibiotic concentrations at the intracellular level is one of the main mechanisms by which bacteria develop antibiotic resistance. The management of efflux pumps, specifically NorA, which is expressed by *S. aureus* strains, is a valuable strategy for restoring susceptibility in strains resistant to antibacterial agents. In recent years, many studies have focused on searching for natural substances that can reverse efflux pump-mediated resistance in *S. aureus*. Extracts and compounds obtained from plants can be efficient efflux pump inhibitors (EPIs) and represent a potentially patient-friendly strategy for controlling *S. aureus*. In the present study, we evaluated the ability of essential oils, petroleum ether extracts, dichloromethane extract (DCME) and six compounds isolated from the heartwood of *Pilgerodendron uviferum* (Cupressaceae) and two synthetic derivatives to inhibit efflux in NorA pumps in the following three *S. aureus* strains: K2378, which overexpressed the *norA* gene (*norA*++), K1902 (*norA*-deleted, Δ*norA*) and the parental strain, NCTC 8325-4. Efflux activity was evaluated using a fluorometric method that measured the accumulation of the universal efflux pump substrate ethidium bromide (EtBr). Only DCME and the compounds 15-copaenol and *epi*-cubenol inhibited EtBr efflux by K2378. Even the lowest concentration of 15-copaenol exhibited a stronger inhibitory effect than carbonyl cyanide *m*-chlorophenyl hydrazone on EtBr efflux by K2378. 15-copaenal only showed inhibition of EtBr efflux in K2378 cells at 125 μg/mL, but not superior to the control inhibitor and 15-copaenyl acetate exerted no intrinsic EPI activity against K2378. Fractional inhibitory concentration index (FICI) values obtained in the checkerboard assays, indicated that all combinations between DCME, *epi*-cubenol and 15-copaenol, and tested antibiotics showed a synergistic effect in wild type, *norA*^++^ and Δ*norA* strains. Moreover, those were not toxic for the HeLa cell line at concentrations in which the synergistic effect and inhibitory activity of efflux pumps was determined. Other extracts and compounds obtained from *P. uviferum* did not display EtBr efflux-inhibiting activity against the evaluated *S. aureus* strains.

## Introduction

Antibiotic resistance is one of the most pressing health problems worldwide due to the continuous appearance of antibiotic-resistant bacterial strains. Antibiotics decrease or attack bacterial pathogens by altering the functions of the bacterial cell wall, proteins, nucleic acids and metabolic pathways. Antibiotic resistance can occur via several mechanisms: (i) the modification of the active site of the target resulting in reduced drug-binding efficiency, (ii) the direct destruction or modification of the antibiotic drug by enzymes belonging to the microorganism, and (iii) the expression of efflux pumps (Walsh, 2000; Khan and Khan, 2016). Overall, these mechanisms can limit the effectiveness of antibacterial drugs, causing substantial clinical problems. This has increased the cost of treating patients and make it difficult or sometimes even impossible (Astolfi et al., 2017). The bacteria that cause the most major clinical problems are *Klebsiella* and *Enterobacter* species, *Enterococcus faecium, Clostridium difficile, Acinetobacter baumannii, Pseudomonas aeruginosa, Escherichia coli*, and *S. aureus* (Marinelli and Tomazs, 2010; Khan and Khan, 2016).

*S. aureus* is a human bacterial pathogen that belongs to the genus *Staphylococcus* and is characterized by Gram-positive cocci capable of living under aerobic to facultative anaerobic conditions (Reddy et al., 2017). Morphologically, they have a diameter ranging from 0.5 to 1.5 μm and can be found singly or in pairs, tetrads, short chains, and irregular grape-like clusters. Additionally, they are non-motile, non-spore-forming and occasionally may contain a polysaccharide capsule. With regard for their biochemical characteristics, *S. aureus* secretes coagulases, ferments mannitol to lactic acid, and reduces nitrates to nitrites (Karmakar et al., 2016; Reddy et al., 2017). These bacteria are normally found in human microflora at multiple body sites, such as the skin, perineum, nares, axillae, and gastrointestinal tract, in which approximately 18 strains can share a benign or commensal relationship with the host. Nevertheless, under pathogenic conditions, these bacteria are very difficult to treat due to their strong ability to produce virulence factors, rapidly colonize the host and develop drug resistance (Akanbi et al., 2017). In addition, *S. aureus* can assume a pathogenic lifestyle in the host cell after entering via a medical implantation or syringe inoculation under hospital conditions (Hiramatsu et al., 2014).

*S. aureus* is often implicated in a wide array of community and hospital infections, including cutaneous and soft tissue infections, bacteremia, septic arthritis, osteomyelitis and necrotizing pneumonia (Tong et al., 2015; Costa et al., 2016; Reddy et al., 2017). Due to indiscriminate use and its recurrent adaptation to clinical treatments over the last 50 years (Hiramatsu et al., 2014), *S. aureus* is always among the first bacterial species to develop resistance to multiple antibiotics (multiple drug resistance, MDR) (Thurlow et al., 2012), including resistance to β-lactamics, aminoglycosides, macrolides, lincosamides, fluoroquinolones (FQs), chloramphenicol, sulfonamides, streptomycin, and tetracycline (Schindler et al., 2013).

The mechanisms described in *S. aureus* that contribute to multidrug resistance include (i) alteration of the target at which the drug exerts its activity, (ii) mutations and alterations in cell wall and cellular membrane components that reduce drug access, and (iii) the derepression of chromosomal components that encode multidrug resistance efflux pumps (Foster, 2017). Efflux pumps are transmembrane proteins with the capacity to expel or exchange toxic compounds from bacterial cells, thus allowing bacterium survival. They are a self-defense mechanism by which the antibiotic is expulsed from the cell, predisposing the organism to high target-resistance (Jo and Ahn, 2016). The pumps are classified according to their characteristics into five families, the most studied of which are NorA, TetK, and MrsA (dos Santos et al., 2018). Analyses of the *S. aureus* genome have determined that a large number of MDR efflux pumps are encoded by its genes; however, it has been shown that NorA is its predominant efflux pump (Handzlik et al., 2013). It has also been reported that the norA gene, which encodes the carrier protein NorA, is overexpressed in 43% of *S. aureus* strains (Astolfi et al., 2017). NorA belongs to the major facilitator superfamily (MFS) of efflux pumps, which are composed of approximately 400 amino acids that are putatively arranged into 12 membrane-spanning helices, with a cytoplasmic loop between the helices (Santos-Costa et al., 2013). This efflux pump uses the proton motive force to energize the transport of antimicrobial compounds through the membrane via an antiport mechanism. NorA has the capability to efflux diverse drugs, such as hydrophilic fluoroquinolone antibiotics, dyes, such as acridine and ethidium bromide (EtBr); and biocides (Van Bambeke et al., 2006; Zimmermann et al., 2017).

Thus, the management of efflux pumps, specifically NorA in *S. aureus*, presents a valuable strategy for activating viable antibacterial targets with the aim of restoring susceptibility to resistant strains to antibacterial agents.

In recent years, many studies have focused on the search for NorA inhibitors of *S. aureus* of synthetic origin such as riparin derivatives (Costa et al., 2016) and 2-phenylquinoline derivatives (Felicetti et al., 2018). Moreover, natural substances derivates from plants have been able to inhibit NorA, such as 5′-methoxy-hydnocarpin isolated from *Berberis fremontii* (Stermitz et al., 2000), baicalein isolated from *Scutellaria baicalensis* (Chan et al., 2011), biochanin A and crysoplenetin isolated from *Artemisia annua* (Tegos et al., 2011), and boeravinone B isolated from the roots of *Boerhavia diffusa* (Singh et al., 2017), among others.

Extracts and compounds obtained from plants, such as efflux pump inhibitors (EPIs), have been shown to exert efficient activity and therefore constitute a friendly strategy for controlling *S. aureus*. Therefore, the present study focuses on the ability of essential oils (EOs), extracts and compounds isolated from the heartwood of *Pilgerodendron uviferum* (D. Don) Florin (Cupressaceae) and synthetic derivatives to inhibit ejection via the NorA efflux pump in a modified strain of *S. aureus, norA++*, which overexpresses the genes that encode the NorA efflux pump.

*P. uviferum* is a conifer native to Chile and Argentina that is known to be highly resistant to attack by microorganisms and insects. It has been postulated that its resistance is related to its chemical composition (Solis et al., 2004; Donoso et al., 2008). Therefore, this tree may be a potential source of natural products that can act as inhibitors of the *S. aureus* NorA efflux pump.

## Materials and Methods

### Essential Oil, Extracts, Sesquiterpenes, and Synthetic Derivatives

#### Isolation and Analyses of Extracts and Sesquiterpenes

Essential oil, light petroleum ether extract (PEE), dichloromethane extract (DCME), natural sesquiterpenes, (-)-*trans*-calamenene (1), cadalene (2), (-)-cubenol (3), (-)-*epi*-cubenol (4), (-)-torreyol (5), and 15-copaenol (6) were obtained from the heartwood of *P. uviferum*. All samples were obtained and analyzed according to methods described in previous studies (Espinoza et al., 2016, 2018).

#### 15-Copaenyl Acetate (6AC) Preparation

15-copaenyl acetate (6AC) was prepared according to Oyarzún and Garbarino (1988), with modifications. Compound 6 (60 mg, 0.273 mmol) was diluted in CH_2_Cl_2_ and treated with acetic anhydride (2.5 mL) and pyridine (0.5 mL) in a 10 mL round-bottom flask at room temperature for 48 h. Then, the reaction mixture was successively extracted with 5% HCl (3 × 5 mL), 5% NaHCO_3_ (3 × 5 mL), and H_2_O (2 × 5 mL). The organic layer was dried with anhydrous sodium sulfate, filtered and evaporated to dryness using a rotatory evaporator to yield a colorless oil corresponding to 15-copaenyl acetate (6AC) (50.6 mg, 0.193 mmol, 70.8%) (Oyarzún and Garbarino, 1988). The NMR data is displayed in Table 1.

**Table 1:** ^13^C (100 MHz, CDCl_3_) and ^1^H-NMR (400 MHz, CDCl_3_) data of 15-copaenol, 15-copaenyl acetate, and 15-copaenal.

Hydrogen and carbon number ^a^	^13^C chemical shift (ppm)			^1^H chemical shift (ppm)		
		
	15-Copaenol (6) ^c^	15-Copaenyl acetate (6AC)	15-Copaenal (6OX)	15-Copaenol (6) ^c^	15-Copaenyl acetate (6AC)	15-Copaenal (6OX)
1 ^b^	44.8	44.7	44.3	1.59 m (1)	1.61–1.57 m (1)	1.58 s (1)
2	50.7	50.9	45.0	1.72 d, *J* = 1 (1)	1.77–1.70 m (1)	2.46 d, *J* = 6 (1)
3	147.6	142.8	151.3	–	–	–
4	118.3	121.7	148.3	5.48 m (1)	5.58–5.14 m (1)	6.73–6.68 m (1)
5	30.3	30.4	32.2	2.26 bs (2)	2.26 bs (2)	2.55 d, *J* = 3 (2)
6	37.6	37.5	37.6	2.12 m (1)	2.14–2.10 m (1)	2.23–2.18 m (1)
7	39.7	39.8	39.3	–	–	–
8	36.4	36.4	35.9	1.73/1.63 m (1)	1.77–1.70/1.63–1.61 m (1)	1.77–1.71 m (2)
9	22.1	22.0	22.1	1.53/1.59 m (1)	1.61–1.57/1.54–1.50 m (1)	1.70–1.60/1.56–1.44 m (1)
10 ^b^	44.9	44.9	44.3	1.69 m (1)	1.70–1.68 m (1)	1.70–1.60 m (1)
11	32.5	32.5	32.5	1.51 m (1)	1.54–1.50 m (1)	1.56–1.44 m (1)
12 ^b^	20.0	19.8	19.8	0.82 d, *J* = 7 (3)	0.83 d, *J* = 7 (3)	0.81 d, *J* = 7 (3)
13 ^b^	20.0	20.0	20.1	0.84 d, *J* = 7 (3)	0.84 d, *J* = 7 (3)	0.84 d, *J* = 7 (3)
14	20.4	20.3	20.3	0.78 s (3)	0.77 s (3)	0.70 s (3)
15	66.4	67.4	191.9	3.98 d, *J* = 1 (2)	4.44 dd, *J* = 7;1 (2)	9.43 s (1)
1’	-	171.4	-	-	-	-
2’	-	21.4	-	-	2.05 s (3)	-


*s, singlet; bs, broad singlet; d, doublet; dd, doublet of doublets; m, multiplet; (No), Integration; *J* (Hz). ^*a*^ Hydrogen assignment was performed using COSY, HSQC-ed, and HMBC data; carbon assignment was performed using DEPT 135 and HSQC-ed data. ^*b*^ Assignment could be exchanged. ^*c*^ Indicates data of 15-copaenol extracted from Espinoza et al. (2018).*

#### Preparation of the Oxidized Derivative 15-Copaenal (6OX)

##### Jones reagent preparation

Chromium (VI) oxide (CrO_3_) (3.5 g, ACS reagent, ≥98%, Sigma-Aldrich, United States) was diluted with H_2_O (5 mL). The mixture was cooled on ice, and 98% H_2_SO_4_ (3 mL) (JTBaker, Avantor, United States) was added at a constant drip. Then, the mixture was diluted with H_2_O (10 mL) and stirred to obtain a solution corresponding to Jones reagent (18 mL) (Meinwald et al., 1973).

##### 15-copaenal (6OX) preparation

15-copaenal (6OX) was prepared according to Harding et al. (1975), with modifications. Compound 6 (60 mg, 0.273 mmol) was diluted in acetone (2.8 mL) and then added to a 10 mL round-bottom flask. The mixture was cooled to 0–5°C in an ice bath, and the Jones reagent was added at a constant drip until the colorless reaction mixture took on an orange color. The mixture was diluted with H_2_O (5.0 mL) and shaken for 10 min. Then, the reaction mixture was extracted with CH_2_Cl_2_ (3 × 5.0 mL). The organic layer was cleaned with an NaCl-saturated solution (3 × 5.0 mL) and evaporated to dryness using a rotatory evaporator. The oily residue was chromatographed on a silica gel column, yielding a pale-yellow oil corresponding to 15-copaenal (6OX) (39.6 mg, 0.182 mmol, 66.6%) (Weyerstahl et al., 1996). The NMR data is displayed in Table 1.

### Analyses of Heartwood EO, Extracts, Sesquiterpenes, and Synthetic Derivatives

The heartwood EO, PEE and DCME and natural sesquiterpenes (1–6) were analyzed according to previously described methods (Espinoza et al., 2016, 2018). The synthetic derivatives 15-copaenyl acetate (6AC) and 15-copaenal (6OX) were analyzed by ATR-FT-IR and ^1^H- and ^13^C-NMR. The FT-IR spectra were recorded on a Perkin Elmer Spectrum 65 spectrometer. Both the ^1^H- and ^13^C-NMR spectra were recorded on a Bruker 400 Ultra Shield spectrometer (Bruker-Biospin GmbH, Germany) with the 5 mm NMR tube, using CDCl_3_ (99.8% D, contains 0.03% (v/v) TMS, Sigma-Aldrich, United States). ^1^H-NMR spectra were acquired using Bruker pulse program with the following settings: flip angle of 30°; relaxation delay (d1) = 0 s, size of fid = 32768, number of scans = 32, spectral width = 14.983 ppm, acquisition time = 5.4657 s, requested probe temperature = 300.0 K. ^13^C-NMR spectra were acquired using Bruker pulse program with the following settings: flip angle of 30°; relaxation delay (d11) = 30 ms and (d12) = 20 μs, size of fid = 16384, number of scans = 24166, spectral width = 240.049 ppm, acquisition time = 0.6783 s, requested probe temperature = 300.0 K. All data acquisition and processing were done with MestreNova 6.0.2 (Mestrelab Research, S.L., Santiago de Compostela, Spain).

### Bacterial Strains and Culture Conditions

The *S. aureus* strains used in this study were an NCTC 8325-4 parental strain, a K1902 mutant (Δ*nor*A), and a K2378 mutant that overexpressed *norA* (*norA*++) from a multicopy plasmid (Felicetti et al., 2018). This strain was produced by cloning *norA* and its promoter into plasmid pCU1 and then introducing the construct into SA-K1902 (Augustin et al., 1992). The bacterial strains were donated by Dr. Glenn Kaatz of the Division of Infectious Diseases, School of Medicine, Wayne State University, United States, and were stored at the Laboratorio de Microbiología Básica y Aplicada, Universidad de Santiago de Chile. Bacterial cultures were grown in Mueller Hinton (MH) broth (Sigma Aldrich, United States) and Luria-Bertani (LB) (Sigma-Aldrich, United States) medium at 37°C with agitation (220 rpm). *S. aureus* K1902 and *S. aureus* K2378 cultures were supplemented with 10 μg/mL chloramphenicol.

### Minimum Inhibitory Concentration (MIC) Determination

The minimal inhibitory concentration (MIC) was defined as the lowest concentration of a compound for which no growth was observed. The MIC determination assays were performed as established by the Clinical and Laboratory Standards Institute [CLSI] (2005) guidelines. The twofold standard micro broth dilution method was performed in 96-well plates. Briefly, bacterial strains were incubated overnight in 3 mL of MH broth at 37°C with shaking at 220 rpm. The bacterial cultures were then diluted in phosphate-buffered solution (PBS) to McFarland 0.5 (1 × 10^8^ CFU/mL). To each well, 188 μL of MH broth, 10 μL of the EO, extract or compound (7.81–1,000 μg/mL), and 2 μL of a bacterial suspension were added to McFarland 0.5 to achieve a final volume of 200 μL. In addition, some wells were used as solvent and sterility controls. The plates were incubated at 37°C for 24 h, and the optical density was measured at 600 nm (OD_600_) in an Elisa lector (Thermo Scientific Multiskan FC Model, Waltham, MA, United States). The MICs of EtBr, a universal substrate of expulsion pumps, and carbonyl cyanide m-chlorophenyl hydrazone (CCCP) were used as the controls for EPIs and determined by the same method. All results are expressed as μg/mL, and experiments were performed in triplicate in three individual experiments.

### Ethidium Bromide Accumulation Assay

The effects of extracts and compounds obtained from *P. uviferum* on the accumulation of EtBr was evaluated in the *S. aureus* NCTC 8325-4 (wild type), K2378 (*norA*++) and K1902 (Δ*norA*) strains. Sub-inhibitory concentrations (½, ¼, and ⅛ of the MIC) of the study extracts and compounds were used in all experiments, and CCCP was used as an inhibitor control. The assay was carried out according to Paixão et al. (2009). Briefly, the *S. aureus* strains were grown in 10 mL of LB medium until they reached a mid-log phase, which corresponded to an OD_600_ of 0.6. Then, the bacteria were centrifuged at 10,786 × *g* for 3 min, the pellet was washed twice with the same volume of PBS, and the OD_600_ of the cellular suspension was adjusted to 0.3. Glucose was added to the cellular suspension to achieve a final concentration of 0.6% (v/v), and aliquots of 95 μL were transferred to 0.2 mL wells in 96-well microtiter plates. Aliquots containing 0.4 μL of EtBr or 0.2 μL of CCCP were then added to obtain final concentrations of 1.0 μg/mL and 1.25 μg/mL of EtBr and CCCP, respectively. Fluorescence (530/580 nm excitation/detection) was monitored by a microplate reader (BioTek Synergy HT Model, Winooski, VT, United States) at 37°C every 5 min for a period of 1 h. The effects of the extracts and compounds on the accumulation of EtBr was determined under conditions that optimized efflux (i.e., the presence of glucose and incubation at 37°C). All experiments were performed in triplicate.

### Checkerboard Assays

The synergy assays between DCME extract or pure compounds, *epi*-cubenol and 15-copaenol in combination with antibiotics (ciprofloxacin and oxacillin) against *S. aureus* strains (wild type, *norA*^++^, Δ*norA*) were evaluated by the checkerboard method described by Motyl et al. (2005), with minor modifications. Briefly, seven serial, twofold dilutions of extract or pure compounds and antibiotics were prepared. In a 96-well plate, 25 μL of each dilution of DCME, *epi*-cubenol or 15-copaenol was added in each vertical row, and 25 μL of antibiotic dilution was added in each horizontal row. Both, first horizontal and vertical rows were left with only one agent, and the following rows contained a fixed amount of one agent and increasing concentrations of the second agent. In the selection of the range of concentrations, the MICs obtained for each tested agent and tested *S. aureus* strains studied were considered. The extract concentrations used ranged from 1.95 to 125 μg/mL, 0.008 to 2 μg/mL for ciprofloxacin and 25 to 0.05 μg/mL for oxacillin. To each well, 100 μL of MH broth and 10 μL bacterial suspension to McFarland 0.5 (1 × 10^8^ CFU/mL) were added. The plates were incubated at 37°C for 24 h and measured at 600 nm in Elisa lector (Thermos Labsystems Multiskan FC Model). All tests were performed in triplicate, in three different experiments. Fractional inhibitory concentrations (FICs) were calculated using the formula FIC_sample_ = (MIC extract + antibiotic/MIC sample) or FIC_antibiotic_ = (MIC sample + antibiotic/MIC antibiotic). The FIC index (FICI; Garvey et al., 2011) for each combination was calculated by the sum of both FIC values, and results were interpreted as follows: FICI ≤ 0.5 synergic effect, 0.5 < FICI ≤ 4 additive effect and FICI > 4 antagonistic effect (Garvey et al., 2011).

### Cytotoxicity

In order to determine the cytotoxic potential of DCME, *epi*-cubenol (4) and 15-copaenol (6) (active extract and compounds against efflux pumps in *S. aureus*), the cervical cancer cell line HeLa (ATCC CCL-2) was used.

#### Cell Culture

The HeLa cell line was grown in monolayers in Dulbecco’s modified medium (DMEM; Corning, NY, United States) with bovine fetal serum 10% (FBS) supplemented with 100 U/mL penicillin and 100 μg/mL streptomycin (Corning Eagle, United States) in a modified Thermo Scientific air incubator (5% CO_2_ at 37°C). The medium was renewed every 2 days to reach 80% confluence. Then, the cells were transferred to T75 flasks, grown to reach 80% confluence, and finally, the culture was divided into sterile 24-well plates.

#### Cytotoxicity Evaluation

Approximately, 10,000 cells per well in a 24-well plate were seeded in 100 μL of DMEM. The cells were treated separately with eight different concentrations of extract and pure compounds (125–0.5 μg/mL). Simultaneously, growth controls were performed, which consisted of cells incubated with culture medium alone and with 4 μL methanol as solvent control. Finally, treated HeLa cells and the respective controls were incubated for 24 h in 5% CO_2_ at 37°C. All tests were carried out in triplicate. Cell viability was determined by counting live and dead cells after staining with 0.5 μM calcein-AM and 2 μM propidium iodide, for 30 min at room temperature, in darkness. Calcein-AM is a live scoreboard and propidium iodide intercalates into DNA being dead cells cell marker. Cells were visualized at 100× magnification on a Nikon microscope equipped Daipot inverted fluorescent and phase contrast optics. Cells were counted in a phase contrast microscope equipped with fluorescence using the following filters: calcein-AM 510–560 nm (excitation) and 590 nm (emission), propidium iodide 536 nm (excitation) and 617 nm (emission).

### Statistical Analyses

Data were analyzed by one- and two-way ANOVA test and two-sample *T*-test using Statistix 10 (Analytical Software, PO Box 12185, Tallahassee, FL, United States).

## Results

### Chemical Analyses

**FIGURE 1:**
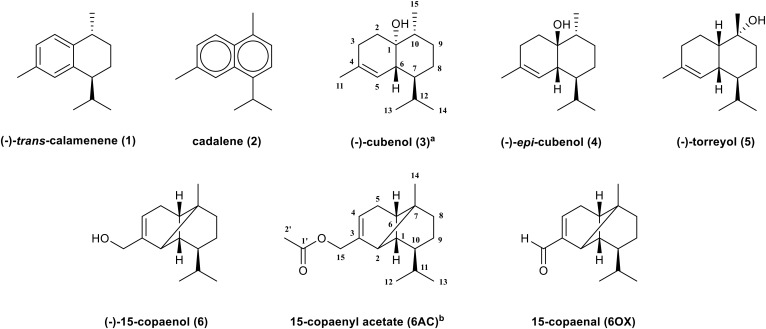
Sesquiterpenes isolated from *Pilgerodendron uviferum* and synthetic derivates of 15-copaenol. ^a^ Numbering belongs to compounds 1–5. ^b^ Numbering belongs to 15-copaenol, 6AC, and 6OX.

The compositions of EO and of PEE and DCME isolated from *P. uviferum* were determined according to Espinoza et al. (2016, 2018), respectively. According to those studies, the isolated EO contained three monoterpenes (0.43%) and 17 sesquiterpenes (86.62%), with (-)-torreyol (5) (24.16%), (-)-cubenol (3) (22.64%), 15-copaenol (6) (15.46%), and δ-cadinene (10.81%) the most abundant components (Espinoza et al., 2016). Regarding the extracts, 19 sesquiterpenes (73.53%) and 6 diterpenes (8.90%) present in the PEE and 17 sesquiterpenes (28.57%) and 5 diterpenes (47.97%) present in the DCME. The sesquiterpenes torreyol (5), cubenol (3), and 15-copaenol (6) were the most abundant compounds in both the PEE and DCME extracts (Espinoza et al., 2018). Sesquiterpenes were obtained from the EO and both of the extracts of *P. uviferum* heartwood using chromatographic methods and characterized by optical rotation, gas chromatography-mass spectrometry (GC/MS), Fourier-transform infrared spectroscopy (FT-IR), and 1D and 2D nuclear magnetic resonance (NMR) experiments. The following sesquiterpenes were identified: (-)-*trans*-calamenene (1), cadalene (2), (-)-cubenol (3), (-)-*epi*-cubenol (4), (-)-torreyol (5), and (-)-15-copaenol (6) (Figure 1).

In MIC determination assays, 15-copaenol (6) had the lowest MIC value between all tested sesquiterpenes. Moreover, in an antibacterial assay against *S. aureus* (ATCC 6538p) and *Bacillus cereus* (ATCC 10876) (Data not published), 15-copaenol exerted the major bacterial growth inhibition. In this context, the structural requirements for antibacterial activity of some terpenoids has been reported. A hydrophobic moiety, consisting of a substituted decalin skeleton, and a hydrophilic region possessing one hydrogen-bond-donor group were the key structural requirements (Urzúa et al., 2008). Then, in order to evaluate the influence of the primary alcohol group in the antibacterial activity of copaene-type sesquiterpenes, hemi-synthetic modifications of 15-copaenol (6) were performed. Thus, 15-copaenol (6) was treated with acetic anhydride in pyridine and yielded 50.6 mg of a colorless oil. The 15-copaenol (6) was also treated with Jones reagent, and the product was chromatographically purified to yield 39.6 mg of a pale-yellow oil. Both synthetic derivatives were analyzed by attenuated total reflectance (ATR)-FT-IR and ^1^H- and ^13^C-NMR (Table 1). The IR spectrum of the acetylated derivate showed very strong CH valence vibration frequencies ranging from 2955 to 2872 cm^-1^; these corresponded to the aliphatic part of the molecule. Additionally, a characteristic tri-substituted C–C double bond stretching vibration valence was observed at 1677 cm^-1^. C–O stretching, C–C(=O)–O asymmetric stretching, and C–C–O asymmetric stretching were observed at 1,740, 1,236, and 1,046 cm^-1^, respectively, and were assigned to ester group vibrations. In contrast to 15-copaenol (6), the acetylated compound produced two additional signals in the ^13^C-NMR spectrum, that corresponded to a quaternary carbon (C-1’) and a methyl carbon (C-2’) at 171.4 and 21.4 ppm, respectively. The C-2’ signal showed a HSQC correlation with a singlet (3H) at 2.05 ppm (H-2’). The IR and NMR spectra indicated the substitution of a hydroxyl group in C-15 of 15-copaenol (6) by an acetyl group (Table 1).

The IR spectrum of the oxidized derivate showed the expected CH valence vibrations and tri-substituted C–C double bond stretching vibration valences from 2,956 to 2,872 cm^-1^ and 1,682 cm^-1^, respectively. Additionally, it showed a carbonyl group band at 1,707 cm^-1^ that corresponded to the C–O stretching of α,β-unsaturated aldehyde. Regarding the NMR analyses, compared with 15-copaenol (6), the methylene group signals from the primary alcohol of 15-copaenol (6) were absent in the oxidized derivate ^1^H and ^13^C-NMR spectrums. However, the C-15 signal at 191.9 ppm was observed in the ^13^C-NMR spectra. The C-15 signal had an HSQC correlation with an unshielded singlet (1H) at 9.43 ppm (H-15) in the ^1^H-NMR spectra. The IR and NMR spectra indicated hydroxyl group oxidation had occurred at C-15 of 15-copaenol (6) to yield an aldehyde group (Table 1). According to the IR and NMR analyses, the synthetic derivatives were identified as 15-copaenyl acetate (6AC), previously synthetized by Oyarzún and Garbarino (1988), and 15-copaenal (6OX), previously synthetized using a different methodology, by Weyerstahl et al. (1996) (Figure 1).

### MIC Determination

The MICs of all extracts and compounds were evaluated in the NCTC 8325-4 (wild type), K2378 (*norA*++), and K1902 (Δ*norA*) *S. aureus* strains. The MICs of the samples are shown in Table 2. In general, MIC values were highest in K2378 (*norA*++) and lowest in K1902 (Δ*nor*A). In the extracts, PEE was the most active, producing MIC values of 31.3 μg/mL for all of the tested *S. aureus* strains. It was followed by EO and DCME, which produced higher values. Regarding the isolated compounds, 15-copaenol (6) was the most active compound against all evaluated strains, with MIC values of 31.3 μg/mL in *S. aureus* wild type and K2378 (*norA*++), respectively, and 62.5 μg/mL in K1902 (Δ*norA*). It was followed by *epi*-cubenol (4) and cubenol (3), which produced values of 31.3 and 62.5 μg/mL, respectively, in wild type *S. aureus*, 62.5 μg/mL in K1902 (Δ*norA*), and 125 μg/mL in K2378 (*norA*++). The other compounds produced no intrinsic antibacterial activity against the *S. aureus* strains (i.e., MIC > 32 mg/L) (Caspar et al., 2015). We found that torreyol (5) was inactive against all evaluated strains, similar to the result reported by Solis et al. (2004), who found that 15-copaenol (6) was more active than cubenol (3) and torreyol (5) against *S. aureus* (ATCC6538p) and *Bacillus subtilis* (ATCC6633).

**Table 2:** MICs of essential oils (EOs), petroleum ether extracts (PEEs), dichloromethane extract (DCME), isolated sesquiterpenes, and synthetic derivates from *P. uviferum* on *S. aureus* strains.

Sample	MIC (μg/mL)		
	
	WT	SA-K1902	SA-K2378
**Extracts**			
EO	62.5	31.3	62.5
PEE	31.3	31.3	31.3
DCME	62.5	62.5	125
**Isolated compounds**			
*Trans*-calamenene (1)	125	125	125
Cadalene (2)	500	62.5	500
Cubenol (3)	62.5	62.5	125
*Epi*-cubenol (4)	31.3	62.5	125
Torreyol (5)	i	i	i
15-Copaenol (6)	31.3	62.5	31.3
**Synthetic derivatives**			
15-Copaenal (6OX)	500	62.5	250
15-Copaenyl acetate (6AC)	125	62.5	500


*WT, wild type (NCTC 8325-4); K1902, *norA*-deleted; K2378, overexpressing *norA*; i, inactive.*

### EtBr Accumulation Assay

**FIGURE 2:**
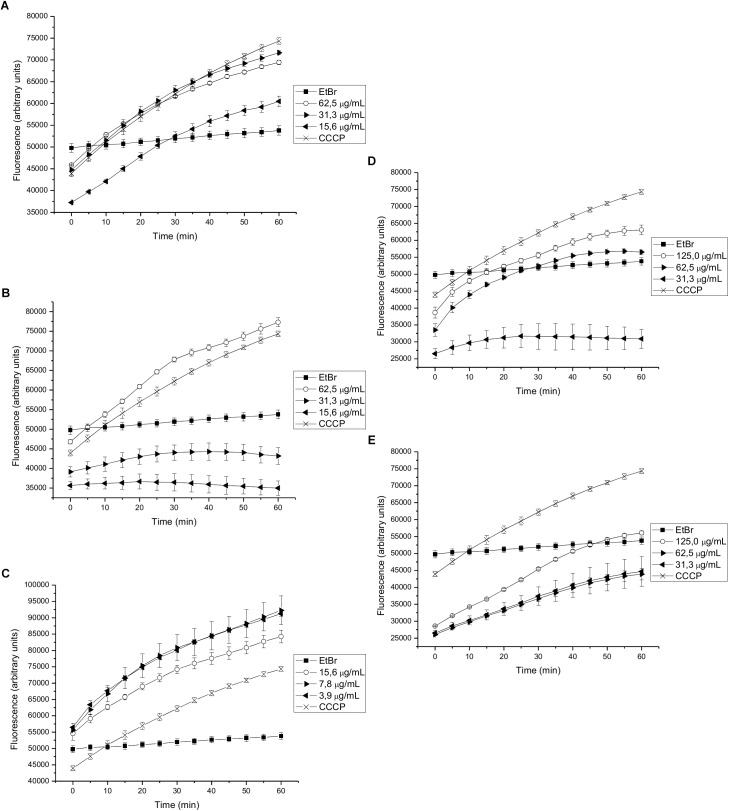
Effect on the intracellular accumulation of ethidium bromide of **(A)** DCME extract, **(B)** *epi*-cubenol (4), **(C)** 15-copaenol (6), **(D)** 15-copaenal (6OX), and **(E)** 15-copaenyl acetate (6AC) in K2378 (*norA++*) strain. The bacteria were exposed to 2.0 μg/mL of EtBr at 37°C for a period of 1 h in the presence of glucose. CCCP (1.25 μg/mL) was used as inhibitor control.

The effects of different sub-inhibitory concentrations of extracts and compounds isolated from *P. uviferum* on EtBr accumulation were next evaluated in *S. aureus* wild type (NCTC 8325-4), K2378 (*norA*++) and K1902 (Δ*norA*) strains. Experiments were carried out using EtBr concentrations that did not affect cell viability. The accumulation of EtBr inside the bacterial cells was increased by the presence of an EPI, such as CCCP, an EPI known to work by acting as a protonophore (Lowrence et al., 2016). Compounds that inhibit the efflux of EtBr results in higher residual fluorescence, whereas non-EPI compounds/untreated cells extrude EtBr, resulting in lower residual fluorescence. Accordingly, only the DCME and 15-copaenol (6) and *epi*-cubenol (4) acted as potential NorA EPIs to induce a higher level of fluorescence over time than was observed for the EtBr curve obtained in untreated K2378 (*norA*++) cells (Figure 2A–C). In particular, 15-copaenol (6) inhibited EtBr efflux in K2378 (*norA*++) cells at all evaluated concentrations (3.9–15.6 μg/mL). Even at the lowest concentration, it displayed a higher level of efflux inhibition than was observed for CCCP (Figure 2C), indicating that it could be indeed a highly potent efflux inhibitor in *S. aureus* (Lowrence et al., 2016). Despite this result, the synthetic derivative, 15-copaenal (6OX) only showed inhibition of EtBr efflux in K2378 (*norA*++) cells at 125 μg/mL, but not superior to the control inhibitor (CCCP) (Figure 2D) and 15-copaenyl acetate (6AC) showed no intrinsic EPI activity against the K2378 strain (Figure 2E). The DCME inhibited EtBr efflux in K2378 (*norA*++) cells at all evaluated concentrations (15.6–62.5 μg/mL) (Figure 2A), but it did not produce better efflux inhibition than was observed for CCCP. *Epi*-cubenol (4) inhibited EtBr efflux in the K2378 (*norA*++) strain only at the highest evaluated concentration (62.5 μg/mL) (Figure 2B); in those experiments, it produced an effect similar to that of CCCP.

**FIGURE 3:**
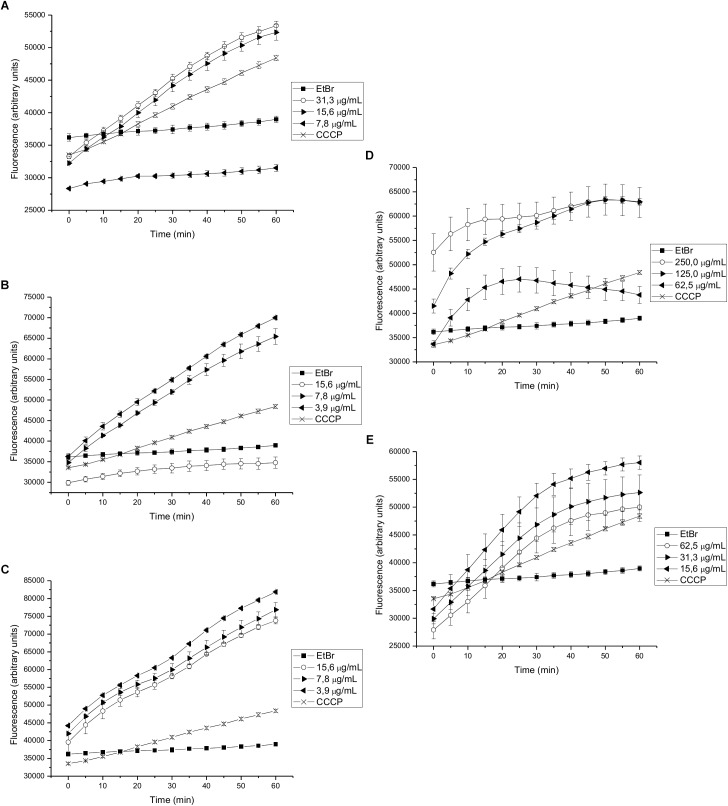
Effect on the intracellular accumulation of ethidium bromide of **(A)** DCME extract, **(B)** *epi*-cubenol (4), **(C)** 15-copaenol (6), **(D)** 15-copaenal (6OX), and **(E)** 15-copaenyl acetate (6AC) in *S. aureus* wild-type (NCTC 8325-4). The bacteria were exposed to 2.0 μg/mL of EtBr at 37°C for a period of 1 h in the presence of glucose. CCCP (1.25 μg/mL) was used as inhibitor control.

**FIGURE 4:**
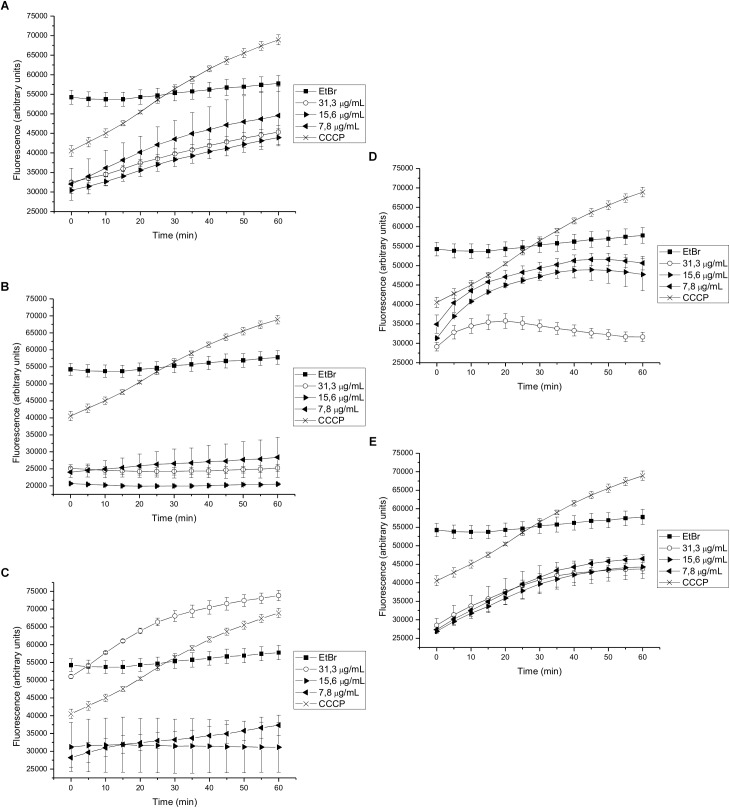
Effect on the intracellular accumulation of ethidium bromide of **(A)** DCME extract, **(B)** *epi*-cubenol (4), **(C)** 15-copaenol (6), **(D)** 15-copaenal (6OX), and **(E)** 15-copaenyl acetate (6AC) in K1902 (Δ*norA*) strain. The bacteria were exposed to 2.0 μg/mL of EtBr at 37°C for a period of 1h in the presence of glucose. CCCP (1.25 μg/mL) was used as inhibitor control.

We found that 15-copaenol (6) also inhibited EtBr efflux in wild type *S. aureus* (NCTC 8325-4) at all evaluated concentrations (15.6–3.9 μg/mL) (Figure 3C), and its synthetic derivatives, 6OX and 6AC, inhibited EtBr efflux at 125–250 μg/mL (Figure 3D) and 15.6–62.5 μg/mL (Figure 3E), respectively. DCME inhibited EtBr efflux in wild type *S. aureus* when added at 15.6 and 31.3 μg/mL (Figure 3A), and *epi*-cubenol (4) inhibited EtBr efflux, better than CCCP, at concentrations of 3.9 and 7.8 μg/mL) (Figure 3B). Additionally, EO and PEE extract inhibited EtBr efflux in wild type *S. aureus* (NCTC 8325-4) (Supplementary Figures 2A,B). Finally, 15-copaenol (6) and EO were the only samples that inhibited EtBr efflux in the K1902 (Δ*norA*) strain (at 31.3 μg/mL and 7.8 μg/mL, respectively) (Figure 4C and Supplementary Figure 3A). Other extracts and compounds obtained from *P. uviferum* did not display EtBr efflux-inhibiting activity in the wild type (NCTC 8325-4), K2378 (*norA*++), and K1902 (Δ*norA*) (Figure 4A,B,D,E) strains of *S. aureus* (Supplementary Figures 1–3).

### Synergy Analysis

The results of synergism are shown in Tables 3, 4. The MICs of DCME, *epi*-cubenol (4) and 15-copaenol (6) varied in a range of 31.3–125 μg/mL in all strains studied. In general, the MIC values of ciprofloxacin were highest in *norA*^++^ (K2378) strain (2 μg/mL) in comparison with Δ*norA* (K1902) strain (0.5 μg/mL), showing ciprofloxacin is substrate of NorA efflux pump. The MIC values of oxacillin did not show significative differences in strains studied (12.5–25 μg/mL). Additionally, was determined that all combinations between DCME extract or pure compounds [*epi*-cubenol (4) or 15-copaenol (6)] with ciprofloxacin or oxacillin decreased MICs values of both antibiotics in tested *S. aureus* strains (Table 3).

**Table 3:** Minimal inhibitory concentration of extract and isolated compounds from *P. uviferum*, and antibiotics and mixtures against *S*. *aureus* strains.

	Minimal inhibitory concentration (μg/mL)									
	
					Antibiotic plus sample					
					
*S. aureus* strains	Antibiotic alone ^a^		Sample alone ^b^		CIP + DCME	CIP + C4	CIP + C6	OX + DCME	OX + C4	OX + C6
WT	CIP	1.0	DCME	62.5	0.125 (8)	0.015 (67)	0.015 (67)	1.5 (8)	1.5 (8)	1.5 (8)
	OX	12.5	EP	31.3						
			15-C	31.3						
K2378	CIP	2.0	DCME	125	0.25 (8)	0.125 (16)	0.25 (8)	3.12 (8)	3.12 (8)	3.12 (8)
	OX	25	EP	125						
			15-C	31.3						
K1902	CIP	0.5	DCME	62.5	0.065 (8)	0.065 (8)	0.031 (16)	1.56 (8)	3.12 (4)	1.56 (8)
	OX	12.5	EP	62.5						
			15-C	62.5						


*WT, wild type (NCTC 8325-4); K1902, *norA*-deleted; K2378, overexpressing *norA*. ^*a*^ CIP, ciprofloxacin; NOR, norfloxacin; OX, oxacillin. ^*b*^ DCME, dichloromethane extract; **C4**, *epi*-cubenol; **C6**, 15-copaenol. ( ), Fold of reducing the MIC of the antibiotic in combination with extract or pure compounds. MIC values are referred to FIC values in Table 4.*

**Table 4:** Fractional inhibitory concentration index (FICI) of different combination between extract or isolated compounds of *P. uviferum* with antibiotics in *S. aureus* strains.

	Fractional inhibitory concentration (FIC) index					
	
*S. aureus* strains	CIP + DCME	CIP + C4	CIP + C6	OX + DCME	OX + C4	OX + C6
WT	0.0307 (S)	0.1403 (S)	0.0773 (S)	0.1437 (S)	0.1561 (S)	0.1903 (S)
K2378	0.1406 (S)	0.1249 (S)	0.1413 (S)	0.1396 (S)	0.1552 (S)	0.1863 (S)
K1902	0.1884 (S)	0.1572 (S)	0.1244 (S)	0.1505 (S)	0.2637 (S)	0.1560 (S)


*FIC index (FICI) were interpreted as: FICI ≤ 0.5, synergic effect; FICI > 0.5 additive effect; FICI > 4 antagonistic effect. (S), synergistic interaction. DCME, dichloromethane extract; **C4**, *epi*-cubenol; **C6**, 15-copaenol.*

The largest reductions in the MIC value of ciprofloxacin were observed when it was mixed with *epi*-cubenol (4) and 15-copaenol (6), reaching 67-fold reduction of MIC values in wild type strain. While the combinations between DCME, *epi*-cubenol (4), 15-copaenol (6) and oxacillin reduced the MIC of this antibiotic 4–16 times fold. Fractional inhibitory concentration index (FICI) values obtained in the checkerboard assays were in the range of 0.0307–0.2637, indicating that all combinations between DCME, *epi*-cubenol (4) or 15-copaenol (6) and tested antibiotics have a synergistic effect (FICI ≤ 0.5) in wild type, *norA*^++^ and Δ*norA* strains (Table 4).

### Cytotoxicity Assays

Toxicity of DCME extract, *epi*-cubenol (4) and 15-copaenol (6) was evaluated due to the synergism they showed with ciprofloxacin and oxacillin, reducing the MIC of the antibiotics until 67-fold in the NCTC-8325-4 strain. In addition, DCME, *epi*-cubenol (4) and 15-copaenol (6) showed activity against efflux pumps in *S. aureus*.

**FIGURE 5:**
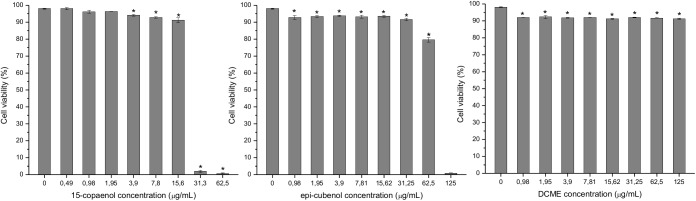
Effects of 15-copaenol, *epi*-cubenol, and DCME extract on HeLa cell viability. The HeLa cells were incubated with different concentration of samples in a range of 125–0.5 μg/mL. Results are the mean ± SE, *n* = 3. The asterisk indicates significantly different from the control at the same level (*P* < 0.05).

The toxicity evaluation of DCME, *epi*-cubenol (4) and 15-copaenol (6) on HeLa cells is shown in Figure 5. When HeLa cell was treated with different concentrations of DCME (125–0.98 μg/mL; it was started from the MIC), the cell viability decreased in 7% compared to the control (98% cell viability), with cell viability percentages in a range of 92.3–91.1 in all concentrations tested. Whereas, when the HeLa cells were treated with the highest concentrations of *epi*-cubenol (4) (125 and 62.5 μg/mL), the viability percentages were reduced to 0.9 and 79.6%, respectively, compared to the control (98.3%). Nevertheless, low concentrations (31.3–1 μg/mL) increases cell viability up to 93.8%. 15-Copaenol showed the greatest toxicity at 62.5 μg/mL (MIC value) and 31.3 μg/mL, and the cell viability decreased by 0.7% and 2%, respectively. By decreasing the concentration to 15.6 and 3.9 μg/mL, the cell viability increased up 94.1%. Finally, using 15-copaenol (6) in concentrations of 1.95–0.49 μg/mL, the viability of HeLa cells increased, respectively, to 96.3% and 98%, significantly equal to the control.

## Discussion

Extracts and compounds were evaluated as bacterial growth inhibitors against wild type, *norA*-deleted, and *norA*-overexpressing strains of *S. aureus*. According to Duarte et al. (2007); Wang et al. (2008), and Oliveira et al. (2013) plant extracts can be classified as antibacterial agents according to their MIC values; MIC < 500 μg/mL indicate strong inhibitors, MIC between 600 and 1,500 μg/mL indicate moderate inhibitors, and MIC > 1,600 μg/mL indicate weak inhibitors. Therefore, the EO and extracts from *P. uviferum* heartwood can be classified as strong inhibitors showing MIC range of 31.3–500 μg/mL in all evaluated *S. aureus* strains. Additionally, the analyses showed that the MIC values were the lowest in the K1902 (Δ*norA*) strain. Wild type and K2378 (overexpressing *norA*) *S. aureus* possessed high levels of antimicrobial resistance because they were capable of reducing the intracellular antibacterial agent concentration via efflux, which was partly mediated by NorA pumps. These pumps were absent in K1902 norA-deleted (Δ*norA*) cells.

In this study, the efflux pump activity was evaluated using a fluorometric method that measured the accumulation of the universal efflux pump substrate EtBr. Overexpressing efflux pumps is an important mechanism by which bacteria can evade the effects of antimicrobial agents. The management of efflux pumps is a valuable strategy to restore susceptibility in strains resistant to antibacterial agents. In this context, DCME and the compounds 15-copaenol (6) and *epi*-cubenol (4) acted as NorA EPIs, as shown by the finding that they produced an higher level of fluorescence over time than was observed for the EtBr curve obtained in untreated *S. aureus* strains. Moreover, 15-copaenol (6) was the only sample that was able to increase the EtBr accumulation in the K1902 (Δ*norA*) strain. Because the *norA* gene was suppressed in the K1902 (Δ*norA*) strain, K1902 cells cannot express the NorA efflux pump. However, other authors have demonstrated that K1902 cells were able to express other efflux pumps for which EtBr is a substrate, such as MepA, TetK, and MrsA (Jang, 2016). It should to be noted that, 15-copaenol (6) was able to increase the EtBr accumulation at all the used concentrations in wild type and K2378 *S. aureus* strains while it was able to increase the EtBr accumulation in K1902 only at 31.5 μg/mL. These results can be explained using the cytotoxicity experiments. 15-Copaenol (6) at 31.5 μg/mL was toxic for HeLa cells (Figure 5). Moreover, MIC of 15-copaenol (6) was 62.5 μg/mL in K1902 strain, but, according with cytotoxicity assays (Figure 5), clearly, at 31.5 μg/mL starts to damage membrane of K1902 cells, thus producing an increase in EtBr accumulation. Therefore, it suggests that 15-copaenol (6) at low concentrations likely could inhibit NorA efflux pumps and other efflux pumps, but at high concentrations (≥31.5 μg/mL) 15-copaenol (6) shows a non-specific effect (such as membrane depolarization) thereby resulting toxic for *S. aureus* strains: 8325-4, K2378 and K1902.

By following a structure-activity analysis, the EtBr accumulation observed in the K2378 (*norA*++) strain indicated that the hydroxylated compound 15-copaenol (6) exerted a stronger inhibitory effect than was found for the tertiary sesquiterpene alcohols *epi*-cubenol (4) and cubenol (3) and the sesquiterpene hydrocarbons *trans*-calamenene (1) and cadalene (2). Among the tertiary alcohols, *epi*-cubenol (4), which has a C1-α-OH, was more active than the C1-epimer, cubenol (3), which was inactive at all evaluated concentrations. Additionally, when the primary hydroxyl group of 15-copaenol (6) was replaced with an aldehyde or an ester group, resulting in the synthetic derivatives 6OX and 6AC, respectively, the modified derivatives did not act like a NorA EPI, indicating that the primary hydroxyl group is essential to generating the EtBr efflux inhibition of copaene-type sesquiterpenes in *S. aureus* K2378 cells. Consistent with these results, Vieira et al. (1991) observed a decrease in the antibacterial activity of 15-copaenol (6) when it was oxidized at C11 and C8 to aldehyde and ketone groups, respectively. Moreover, the MIC values obtained in this study (Table 2) are consistent with the results of the structure-antibacterial activity analyses performed in wild type and K2378 *S. aureus*. However, when we studied growth inhibition in the K2378 strain, we found that the MIC values for cubenol (3) and *epi*-cubenol (4) were the same. These data indicate that the C1-tertiary hydroxyl configuration was not involved in antibacterial activity. This analysis represents an initial approach to determining the structural characteristics of the cadalene-type sesquiterpenes that act to induce NorA efflux pump inhibition in *S. aureus* strains.

The results obtained in synergism assays, are coincident with those previous *in vitro* studies which showed a synergistic effect between plant extracts and/or isolated compounds with different classes of antibiotics against sensitive and multidrug resistant pathogenic strains. It has also been determined that these combinations reduced the minimum inhibitory concentration of antibiotics in different *S. aureus* strains (Chung et al., 2011; Langeveld et al., 2014; Bag and Chattopadhyay, 2015; Dong et al., 2017). In addition, Magi et al. (2015), described that carvacrol, the major component of the EOs of *Origanum* and *Thymus* plants act in synergism with erythromycin against 21 of 32 clinical erythromycin-resistant Streptococci, with reduction of the erythromycin MIC up to 2,048-fold.

Hemaiswarya et al. (2008); Wagner and Ulrich-Merzenich (2009), and Wagner (2011) have described four mechanisms by how antibiotic synergism with phytoextracts occurs: (a) multi objective effect, which is based on the fact that the action of the phytoextract affect several cellular targets, such as enzymes, receptors and substrates, among others; (b) pharmacokinetic effects based on the fact that components of the phytoextract help the solubility; (c) elimination or neutralization of toxic or adversely acting substances by one agent that has been added to an extract; (d) the interaction of the components of the phytoextract with the resistance mechanisms of the bacteria. Our results showed that DCME extract, *epi*-cubenol (4) and 15-copaenol (6) can act in synergy with antibiotics with different mechanisms of action. The ciprofloxacin (a fluoroquinolone) has as cellular target the DNA gyrase, enzyme involved in the synthesis of DNA, while oxacillin (a β-lactam) is involved in the inhibition of the peptidoglycan synthesis (synthesis of the cell wall). Then, these antibiotics in combination with extracts and pure compounds resulting in a multi-objective effect. However, the synergistic effect can be produced by more than one mechanism, for example a multi-objective effect and the inhibition of bacterial resistance mechanisms such as efflux pumps.

Moreover, according to ISO 10993-5 (2009), DCME extract, *epi*-cubenol (4), 15-copaenol (6) were not toxic for the HeLa cell line at concentrations ≤125 μg/mL, ≤62.5 μg/mL, and ≤15.6 μg/mL, respectively, in which the synergistic effect and inhibitory activity of efflux pumps was determined in *S. aureus* strains. However, cytotoxicity studies of sesquiterpenes are scarce. (+)-Torreyol (5) has showed low cytotoxicity (Yap et al., 2017), and cubenol (3) and cadalene (2) are not cytotoxic (Kouamé et al., 2013; Jiangseubchatveera et al., 2015).

Other terpenes are also capable of producing efflux pump inhibition. Oluwatuyi et al. (2004) demonstrated that the abietane diterpenes, carnosic acid and carnosol, which were isolated from *Rosmarinus officinalis*, had modest EPI activity. Carnosic acid decreased the MIC values of erythromycin against RN4220 (pUL5054; *msrA*++) by eightfold and caused a twofold decreased in the EtBr MIC against the SA-1199B strain of *S. aureus*, which overexpresses the *norA* gene that encodes the NorA MDR efflux pump (Sun et al., 2016), and that of tetracycline against the XU212 strain of *S. aureus*, which possesses the TetK tetracycline efflux protein (Sun et al., 2016). However, carnosic acid inhibited EtBr efflux in SA-1199B cells, suggesting that it has weak NorA-inhibitory activity. Carnosol was less active than carnosic acid, but when used in combination with tetracycline, it produced a fourfold decrease in the MIC against XU212 cells. The authors mentioned that high diterpene lipophilicity is a unique common characteristic of these factors and concluded that lipophilicity was essential to efflux pump activity in multi-resistant Gram-positive bacteria. However, in the present study, we found that the lipophilic sesquiterpenes *trans*-calamenene (1) and cadalene (2) were not NorA EPIs and that these isolates resulted in the highest MIC values in all of the evaluated strains. This finding suggests that other structural factors are needed to produce NorA efflux pump inhibition.

Ferruginol, a diterpene isolated from *Chamaecyparis lawsoniana*, was demonstrated to have an IC_50_ of 17 μM in an EtBr efflux-inhibition assay performed using SA-1199B cells (Yusa and Tsuruo, 1989). Both reserpine and ferruginol caused a fourfold reduction in the MIC of tetracycline in XU212 cells. Ferruginol also increased the activity of erythromycin by fourfold against *S. aureus* RN4220 cells (pUL5054; *msrA*++), suggesting that these cells exhibit a rarely observed MsrA inhibitory activity (Smith et al., 2007a). It should be noted that ferruginol and other similar compounds, such as 6,7-dehydroferruginol, hinokione, hinokiol, and sugiol, were present in the DCME and PEE isolated from *P. uviferum*. These compounds could be related to the inhibitory activity of DCME. Totarol, a phenolic diterpene isolated from *Chamaecyparis nootkatensis*, reduced by eightfold the MIC of EtBr against SA-K3092 (*norA*++); EtBr efflux was similarly inhibited by reserpine (Smith et al., 2007b). Interestingly, this concentration of totarol mediated an eightfold reduction in the erythromycin MIC in RN4220 cells (pUL5054), demonstrating that it exerts activity against MsrA (Schindler et al., 2013).

Currently, other types of secondary metabolites have also been suggested to act as EPIs in Gram-positive bacteria. Reserpine (an indole alkaloid), flavones, isoflavones, flavolignans, coumarins, galvanic acid (sesquiterpene-coumarin), porphyrins, and chalcones, among others, have been described as EPIs (Markham et al., 1999; Gibbons et al., 2003; Stavri et al., 2007; Smith et al., 2007a; Schindler et al., 2013; Hazra et al., 2018). Unfortunately, the substantial structural heterogeneity of those secondary metabolites has not allowed a correlation analysis of the structural requirements of efflux pump inhibition. In addition, none of these potential EPIs has been approved for clinical use, partly because their inhibitory mechanisms have not been clarified (Sun et al., 2014). Future research should include detailed structure-activity relationship (SAR) studies, which today are very scarce, as the results of such studies could lead researchers to discover new EPIs.

## Author Contributions

JE, AU, LS, and MWi designed the research. JE and AU analyzed chemical data. LS and MWa performed bioassays. PM performed the cytotoxicity test. LS, MWa, and JE performed statistical analyses. JE, AU, LS, PF, and LM wrote the manuscript. All authors read and approved the final manuscript.

## Conflict of Interest Statement

The authors declare that the research was conducted in the absence of any commercial or financial relationships that could be construed as a potential conflict of interest.
