# Supplementary figures and images for: Essential Oil, Extracts, and Sesquiterpenes Obtained From the Heartwood of Pilgerodendron uviferum Act as Potential Inhibitors of the Staphylococcus aureus NorA Multidrug Efflux Pump
Source: Front Microbiol. 2019 Feb 26;10:337. doi: 10.3389/fmicb.2019.00337 (PMC6400098; doi:10.3389/fmicb.2019.00337)

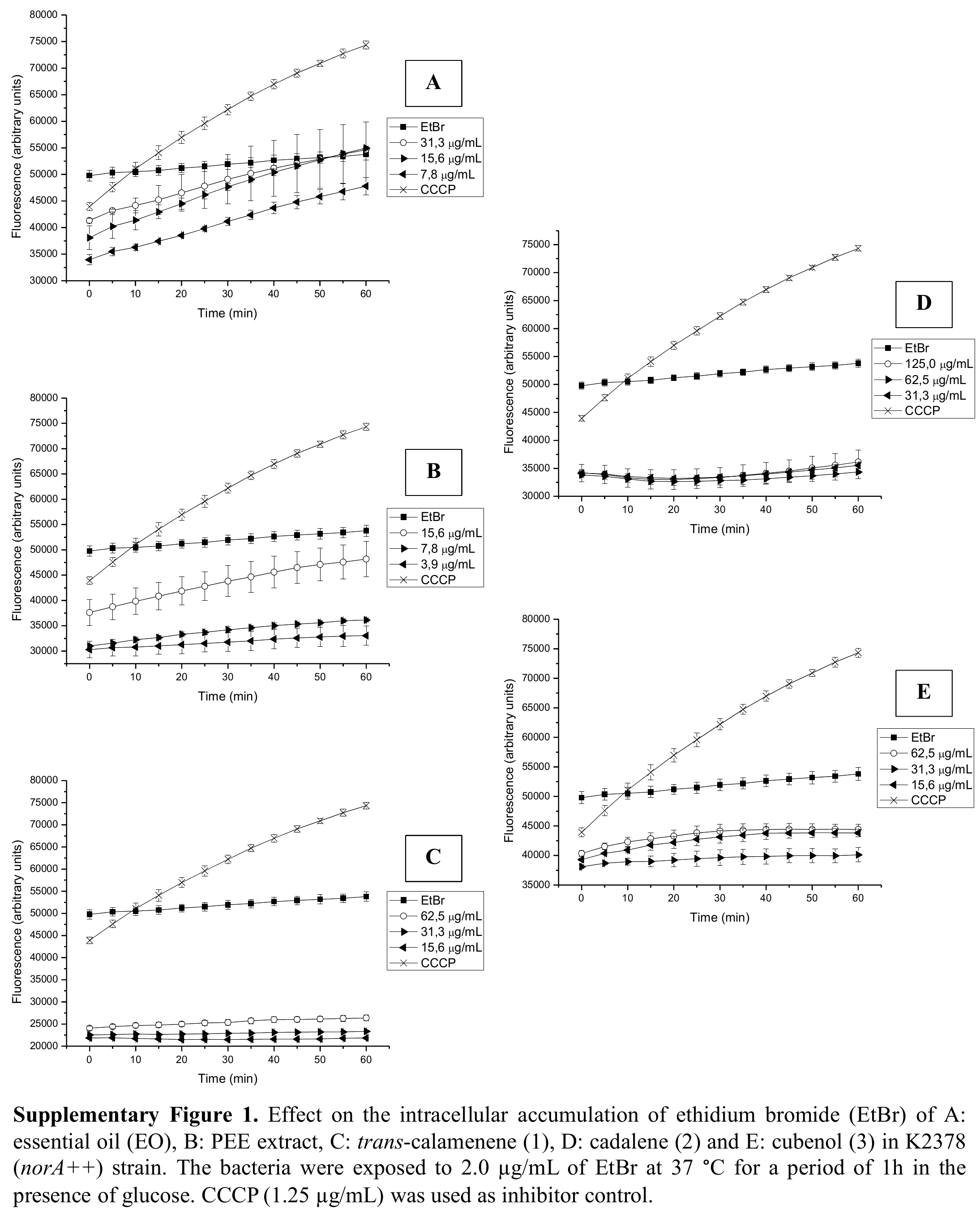

Supplement: Supplementary file 1 [file Image_1.TIF]

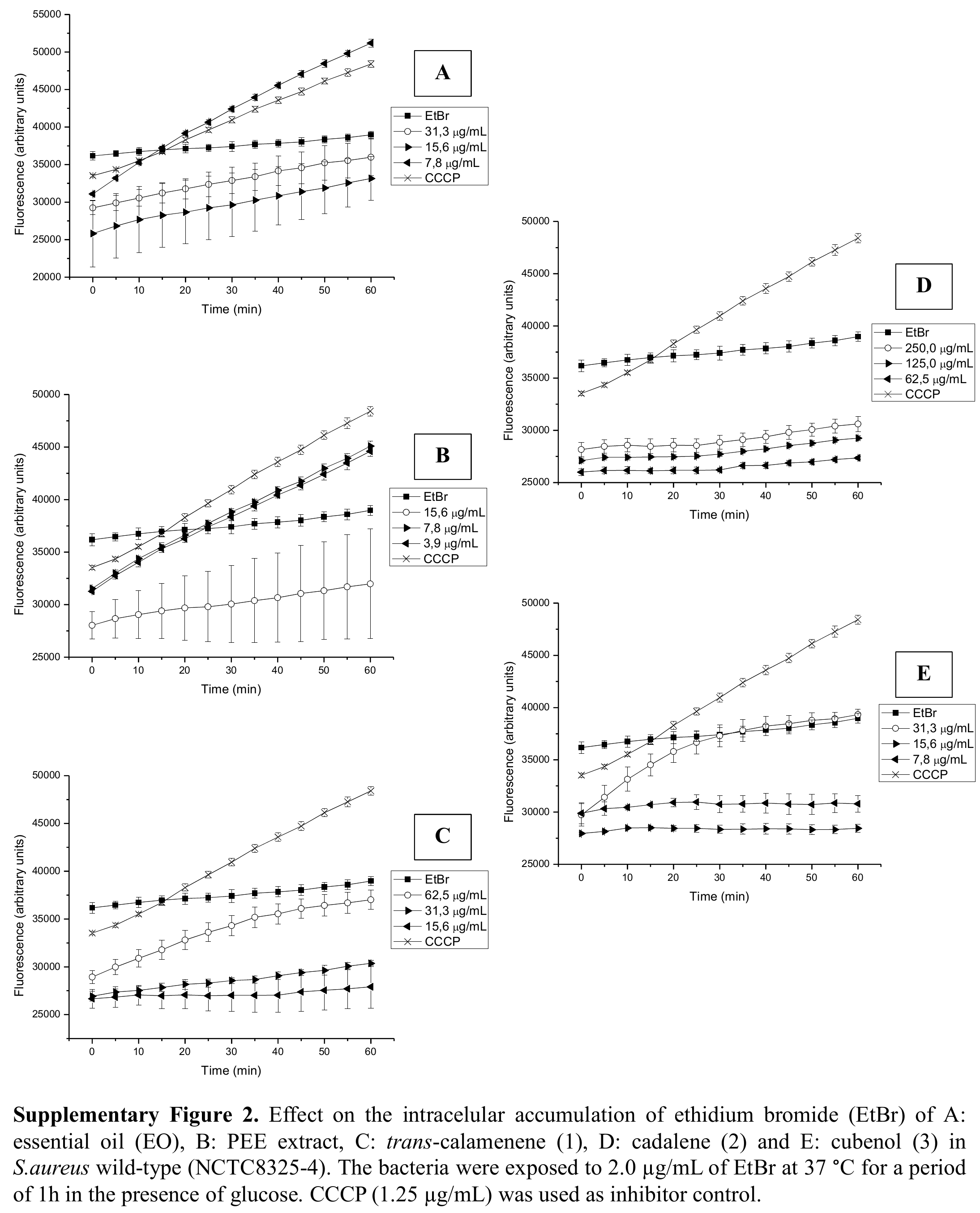

Supplement: Supplementary file 2 [file Image_2.TIF]

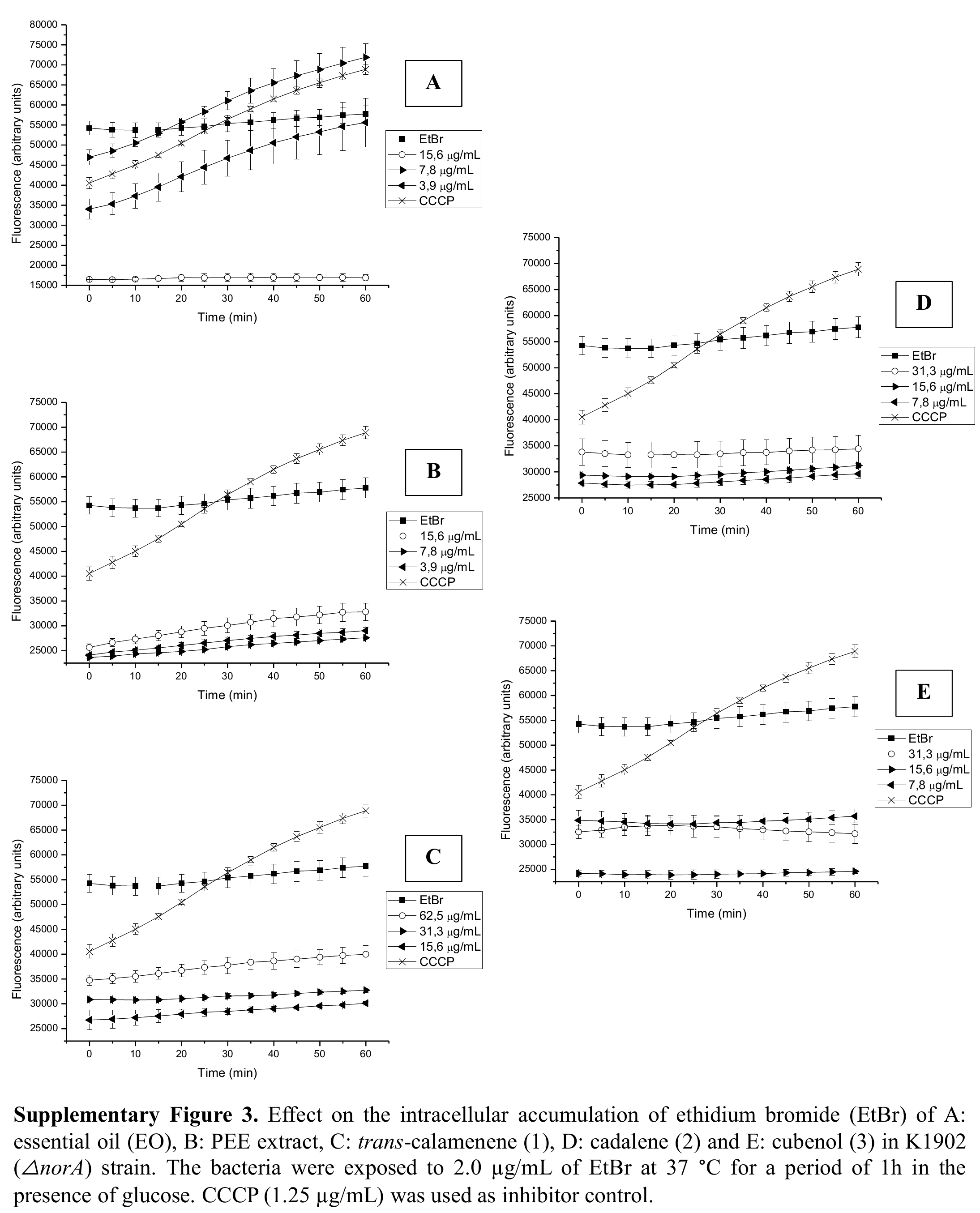

Supplement: Supplementary file 3 [file Image_3.TIF]
